# An Innocent Appearing Subcutaneous Nodule Diagnoses a Small Cell Lung Cancer in a Never-Smoker Female

**DOI:** 10.1155/2014/268404

**Published:** 2014-03-10

**Authors:** Nupur Sinha, Masooma Niazi, Gilda Diaz-Fuentes, Richard Duncalf

**Affiliations:** ^1^Division of Pulmonary and Critical Care Medicine, Bronx Lebanon Hospital Center, Albert Einstein College of Medicine, Bronx, NY 10457, USA; ^2^Department of Pathology, Bronx Lebanon Hospital Center, Albert Einstein College of Medicine, Bronx, NY 10457, USA

## Abstract

Lung cancer among never-smokers is recognized as the 7th most common cause of cancer death globally. Adenocarcinoma is the most commonly reported histology. Small cell lung cancer (SCLC) has the strongest association with smoking and is rarely reported in never-smokers. Although lung cancer in never-smokers is more common in women, the overall incidence of SCLC in female never-smokers still remains low. Soft tissue metastases from any cancer are rare with an overall prevalence of 1.8%. Soft tissue metastases from lung primary are uncommon, mostly from adenocarcinoma, and portend a poor prognosis. Cutaneous metastases from SCLC are exceptionally rare with reported incidence of 0.3% to 0.8%. We believe ours is the first reported case of SCLC presenting as subcutaneous nodule, in a never-smoker, otherwise asymptomatic female. The diagnosis of SCLC was made incidentally by the excisional biopsy of the subcutaneous nodule. Subsequent CT chest and PET scan revealed a hypermetabolic right lower lobe spiculated lung mass with adrenal and liver involvement. Platinum and etoposide chemotherapy with prophylactic cranial irradiation was initiated for advanced SCLC, and she required further irinotecan and taxol for subsequent pancreatic and adrenal metastases. With continued deterioration, she died approximately 36 months from diagnosis, while under hospice care.

## 1. Introduction

Lung cancer in never-smokers is increasingly being recognized as a distinct entity and ranks as the seventh most common cause of cancer death globally [1, 2]. Worldwide, 15% of men and 53% of women with lung cancer are never-smokers [2]. Adenocarcinoma is the most commonly reported histology in never-smokers [1, 2]. Recognized as an entity distinct from other lung cancers in 1926 by Dr. W. G. Bernard, small cell lung cancer (SCLC) accounts for 15% of annual lung cancers in the USA and is known to have the strongest association with tobacco use. More than 95% occur in smokers, with 95% fatality [3]. Small cell lung cancer in never-smokers is rarely reported. Soft tissue metastases from lung cancer are uncommon with reported overall prevalence of 2.3% [4], and rarely reported from SCLC. We report the first case of SCLC in a never-smoker woman presenting as subcutaneous nodule.

## 2. Case Presentation

A 54-year-old woman presented with a two-month history of an enlarging, slightly painful left flank nodule. There was no preceding history of trauma or insect bite to the involved region. She denied fever, chills, rash, cough, shortness of breath, hemoptysis, mouth ulcers, arthralgias, dysuria, or loss of weight. Her medical history included chronic anemia, treated latent TB, cervical dysplasia, and hysterectomy. She denied tobacco use, second hand smoking, or occupational exposure. Family history was significant for various cancers: bone cancer in her father, unknown facial cancer in a brother, liver cancer in an uncle, and a brain tumor in an aunt. Physical exam revealed only a single 2 × 2 cm firm, slightly tender, freely mobile, nonfluctuant, left flank mass without induration, erythema, or involvement of the skin. No other nodules, masses, or lymphadenopathy was found. Laboratory demonstrated a mildly elevated erythrocyte sedimentation rate at 35 mm/hr and a lactate dehydrogenase of 220 IU/L. Autoimmune, HIV, and hepatitis work ups were negative, and thyroid function was normal. A chest X-ray (CXR) and subsequent whole body computerized axial tomography (CT) scan done three years earlier as part of an anemia work up were unremarkable with no evidence of lymphadenopathy or malignancy.

**Figure 1:**
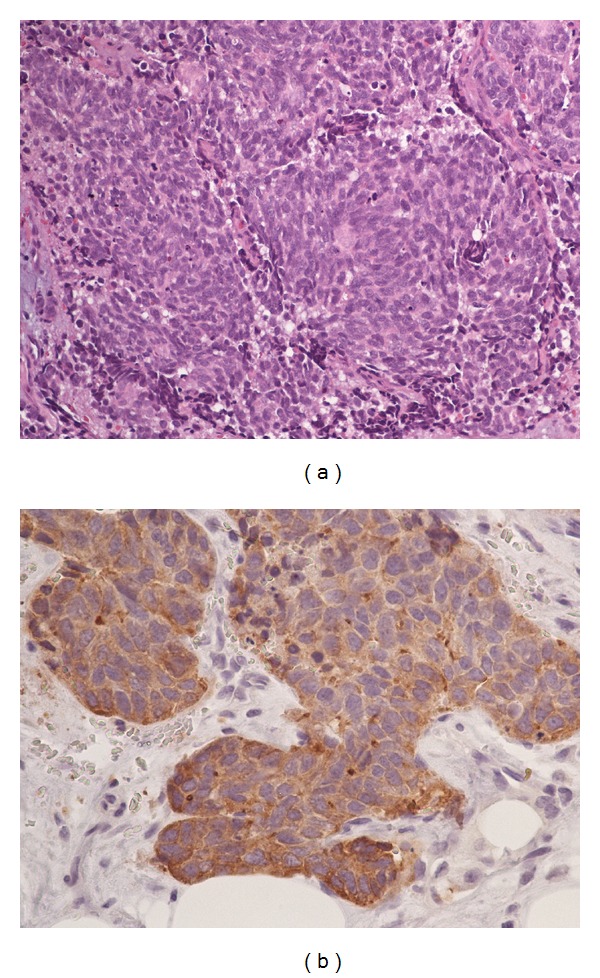
(a) Subcutaneous tissue with neuroendocrine carcinoma (small cell type) composed of sheets of small spindle cells with finely granular chromatin and mitoses. (b) Tumor cells immunoreactive to chromogranin A.

**Figure 2:**
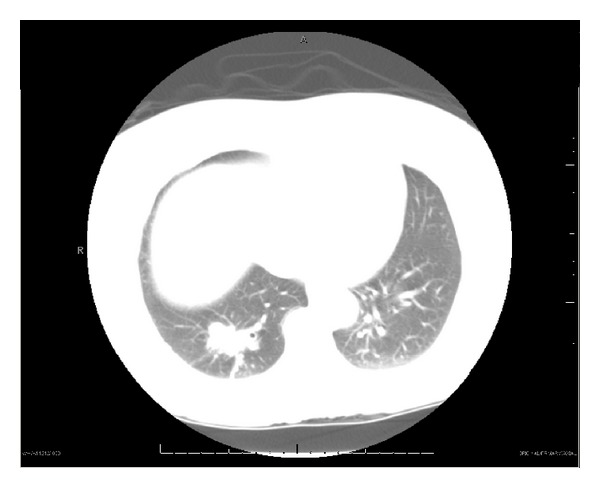
CT chest: right lower lobe spiculated mass.

With a presumptive diagnosis of neurolipoma, the patient underwent excision with wide margins. Operative findings were significant for reddish brown appearing 2 cm firm, subcutaneous mass superficial to the fascia, surrounded by fat. Histopathology revealed a small cell type neuroendocrine tumor. Immunohistochemical stain favored lung primary, with staining positive for TTF-1, CD56, synaptophysin, and chromogranin A (Figure 1). Subsequent CXR and CT demonstrated a 3.4 cm right lower lobe spiculated mass, distal atelectasis, and ipsilateral hilar lymph nodes, the largest being 17 mm (Figure 2). Positron emission tomography (PET) scan confirmed a hypermetabolic right lower lobe lung mass with hilar adenopathy and possible liver and adrenal involvement. In view of sufficient diagnostic evidence, further invasive workup was not pursued. Platinum and etoposide chemotherapy was initiated for advanced SCLC. Despite prophylactic cranial irradiation, she developed cerebral metastasis requiring further radiotherapy. Irinotecan and taxol chemotherapy were required for subsequent pancreatic and adrenal metastases. She continued to maintain a good functional status for the most part despite recurrent metastases and died 36 months from diagnosis, while under hospice care.

## 3. Discussion

The differential diagnosis of a subcutaneous nodule is extensive and mostly benign, including traumatic, infectious, inflammatory, and neoplastic etiology. Metastasis to soft tissue, defined as metastasis to skeletal muscle, skin, and subcutaneous tissues, has only rarely been reported [4]. The available literature does not distinguish between cutaneous and subcutaneous metastases. While earlier studies investigating the prevalence of cutaneous metastases from any cancer have reported an overall incidence of 0.75%–9% [4, 5], a more recent study on 500 patients with cancer reported soft tissue metastases in 1.8% of cases [5]. The most common primary cancers associated with cutaneous metastases are lung and colon in males and breast in females [6].

Cutaneous metastases with a lung primary are relatively uncommon and portend a poor prognosis. Skin metastases are seen in 1 to 12% of patients with lung cancer during their life time [7], with up to 24% of these presenting with a cutaneous lesion upon initial presentation [8]. Common sites of metastases include the chest, back, abdomen, head, and neck [5, 8]. Adenocarcinoma has been shown to be the histological variant of lung cancer most commonly associated with soft tissue metastasis [8]. Cutaneous metastases from SCLC are exceptionally rare with reported incidence of 0.3% to 0.8% [9, 10].

SCLC has been historically described to be extremely rare in female never-smokers. More recent studies have revealed that lung cancer in never-smokers is more common in women [1, 2, 11]. The overall incidence of SCLC in female never-smokers still remains low accounting for approximately 2.9% of all female patients diagnosed with lung cancer [11]. Genetic predisposition has been studied as an important risk factor in never-smokers with lung cancer, although reported incidence remains rare at 1% with more than 3 affected relatives [12].

Chemotherapy is the standard therapeutic modality for extensive disease, with a median survival of 7–12 months [13]. For extensive stage SCLC, the role of etoposide and cisplatin is well studied and described in literature. Prophylactic cranial irradiation in chemotherapy-responding patients modestly improves a disease-free and an overall survival and may decrease the risk of developing brain metastases [13]. Patients presenting with skin lesions at the time of diagnosis have been shown to have a lower survival rate than patients who develop skin metastasis later in the disease course. Treatment of solitary skin metastasis includes surgery combined with either or both chemotherapy and radiation [8]. Although studies have shown that females have a higher objective response rate, median survival and 2-year disease-free survival rate compared to males, the overall median survival reported after diagnosis of cutaneous metastasis remains low at 5–7.5 months [11].

Our case presented with a seemingly benign small abdominal wall nodule. Most common benign etiologies were ruled out by history and the absence of systemic complaints made it unlikely to be a manifestation of systemic disease. Although she had strong family history of various cancers, her younger age and prior normal sex and age appropriate screenings placed her at low risk for malignancy.

Only two cases of lung cancer with metastasis to soft tissue in females have been reported, both were NSCLC, smoking status unknown, and neither had soft tissue metastasis as the sole presenting complaint [5]. Literature also describes three cases of lung carcinoids with subcutaneous metastasis [14] and a single case report of SCLC in a female presenting with multiple systemic complaints along with a subcutaneous nodule [8], but they were all smokers and did not have subcutaneous metastasis as the sole presenting complaint. We believe ours is the first reported case of SCLC presenting as subcutaneous nodule, in a never-smoker, otherwise asymptomatic female, diagnosed incidentally by excisional biopsy of the presenting nodule. As noted in earlier reports, the presentation was associated with extensive disease and systemic metastases, and she continued to have recurrent metastases despite standard therapy. Still, her good functional status and unusual survival of approximately 36 months from presentation are remarkable and warrant further studies in such cases.

## 4. Conclusion

Lung cancer in never-smokers is emerging as a distinct clinical entity, with different genetic mutations and response to novel targeted therapies. Subcutaneous metastasis from a primary lung cancer is unusual and ominous. Being more commonly accepted as a disease of smokers, there is a potential for failure or delay in the diagnosis of lung cancer in a young, never-smoker patient presenting with atypical manifestations such as subcutaneous nodules. While there is extensive work ongoing on identifying causative factors other than smoking in nonsmall cell lung cancer (NSCLC) resulting in major therapeutic advances and improved outcome in NSCLC, there is paucity of similar studies in SCLC.

Our case, with an atypical presentation in otherwise asymptomatic and low risk patient, and her remarkable survival after the incidental diagnosis draws attention to the need for studying more closely the causative as well as prognostic associations in this unique subset of population. We recommend reporting rare cases of SCLC in never-smokers for further analysis of potential risk factors and management options. In addition, our case demonstrates how even a single, new subcutaneous lesion can represent serious occult pathology in a patient with low suspicion for internal malignancy, thus warranting a low threshold for biopsy.

## Abbreviations

CT: Computerized axial tomography
CXR: Chest X-ray
HIV: Human immunodeficiency virus
NSCLC: Nonsmall cell lung cancer
PET: Positron emission tomography
SCLC: Small cell lung cancer.
